# Supplementary material for: The acinar differentiation determinant PTF1A inhibits initiation of pancreatic ductal adenocarcinoma
Source: eLife. 2015 Jul 7;4:e07125. doi: 10.7554/eLife.07125 (PMC4536747; doi:10.7554/eLife.07125)
Supplement: Supplementary file 1. — R markdown for RNA-seq analysis. HTML annotation of R software package analysis performed to generate the data and analyses presented in Figure 5. DOI: http://dx.doi.org/10.7554/eLife.07125.022 [file elife07125s001.html]

Krah et al. RNA-seq analysis


# Krah et al. RNA-seq analysis

```
# before proceeding, set working directory to be wherever RNA-seq and other data files are stored

# clear memory
rm(list=ls())
```

Import data files

```
# RNA-seq results, filtered for differentially expressed genes (FDR < 0.05)
dat <- read.table("diff_exp.txt",sep="\t",header=T,stringsAsFactors=F)
head(dat)
```

```
##    Symbol log.FC      FC log.CPM     CPM      P.value          FDR
## 1   S100g  10.26 1224.97    0.22    1.17 1.020000e-17 7.300000e-16
## 2  Slc9a4   8.78  439.22   -1.07    0.47 3.470000e-12 1.120000e-10
## 3    Pga5   6.74  106.78   10.73 1697.60 3.350000e-39 1.810000e-36
## 4 Glycam1   6.66  101.37    0.33    1.26 1.064832e-03 5.725028e-03
## 5    Chia   6.60   96.79    9.35  653.29 2.630000e-35 1.120000e-32
## 6    Fgl1   6.57   94.94    1.20    2.29 2.180000e-25 4.070000e-23
```

```
# gene signatures from: 
# Singh et al., Cell 2009 (http://www.cell.com/cancer-cell/abstract/S1535-6108%2809%2900111-1, Table S1: top 250 RAS addiction-specific probes)
# Loboda et al., BMC Med Genom 2010 (http://www.biomedcentral.com/1755-8794/3/26, Additional File 3)
# All gene symbols updated to most current HGNC nomenclature (via http://www.genenames.org/cgi-bin/symbol_checker)
singh <- scan("singh.txt",what="character",sep="\n")
#collapse to unique symbols
singh <- unique (singh)
loboda <- scan("loboda.txt",what="character",sep="\n")
ras <- unique (c(singh,loboda))

# PDAC subtype signatures from Collisson et al., Nature Medicine 2011
classical <- scan("Collisson_classical.txt",what="character",sep="\n")
exocrine <- scan("Collisson_exocrine.txt",what="character",sep="\n")
```

Convert human genes to mouse, via HomoloGene IDs

```
# species conversion function
convert <- function (symbols,in_spec,out_spec,hom) {
  # symbols = vector of gene symbols from first species
  # in_spec = Taxon ID of first species
  # out_spec = Taxon ID of species to which you wish to convert gene symbols
  # hom = HomoloGene table
  
  hom1 = hom[hom$NCBI.Taxon.ID==in_spec,c("HomoloGene.ID","Symbol")]
  colnames(hom1) = c("HomoloGene.ID","Former.Symbol")
  hom2 = hom[hom$NCBI.Taxon.ID==out_spec,c("HomoloGene.ID","Symbol","EntrezGene.ID")]
  
  matches = toupper(hom1$Former.Symbol) %in% toupper(symbols)
  IDs = hom1$HomoloGene.ID[matches]
  out = hom2[hom2$HomoloGene.ID %in% IDs,]
  
  out2 <- merge (out,hom1,by="HomoloGene.ID")
  
  return (out2)
}

# read mouse-human homology table (downloaded from http://www.informatics.jax.org/homology.shtml)
hom <- read.delim("HOM_MouseHumanSequence.rpt",sep="\t")
ras_mus <- convert (ras,9606,10090,hom)
classical_mus <- convert (classical,9606,10090,hom)
exocrine_mus <- convert (exocrine,9606,10090,hom)

write.table(ras_mus,"ras_mus.txt",sep="\t",row.names=F)
write.table(classical_mus,"classical_mus.txt",sep="\t",row.names=F)
write.table(exocrine_mus,"exocrine_mus.txt",sep="\t",row.names=F)
```

Label classes of significantly changed genes, set up for plotting

```
# add class names to gene sets
ras_mus$Class="RAS"
ras_mus$Plot.order=2
classical_mus$Class="classical"
classical_mus$Plot.order=4
exocrine_mus$Class="exocrine"
exocrine_mus$Plot.order=3
# unique = set of specific genes to mark in volcano plot
unique <- data.frame(c("Sox9","Cpa1","Ptf1a","Nr5a2","Bhlha15"))
unique$Class="unique"
unique$Plot.order=5

sets <- rbind(ras_mus,classical_mus,exocrine_mus)
# get rid of extraneous variables
sets <- sets[c(2,5,6)]

# merge gene set info into RNA-seq data-set
dat2<-merge(dat,sets,all.x=T)

# set class of unlabeled genes to "other"
dat2[is.na(dat2$Class),]$Class <- "other"
dat2$Plot.order[dat2$Class == "other"] <- 1

# order data for plotting
dat2 <- dat2[order(dat2$Plot.order),]
```

Draw pretty plot, suitable for saving to file

```
#read the color palette file, assigns colors to each class
palette <- read.table("palette.txt",sep="\t",header=T,comment.char="",stringsAsFactors=F)

library(ggplot2)
theplot <- ggplot(dat2, aes(x=dat2$log.FC, y=-log10(dat2$FDR), colour=dat2$Class, size=1.5)) + geom_point() + theme_bw(15) + labs(x="log2 fold change", y="-log10 FDR") + scale_color_manual(values=palette$Color[order(palette$Class)], name="Class") + scale_size(guide="none") + coord_fixed(ratio=0.5)

theplot
```

Interrogating enrichment of individual classes

```
# dist = function for returning binomial test results of enrichment of genes in set y among up- vs. down-regulated genes in dataset x

dist <- function (x,y) {
  upreg <- x$Symbol[x$log.FC>0]
  downreg <- x$Symbol[x$log.FC<0]
  upreg.class <- x$Symbol[x$Class == y & x$log.FC>0]
  downreg.class <- x$Symbol[x$Class == y & x$log.FC<0]
  cat("Gene class:",y,"\n")
  cat("Upregulated genes:",length(upreg.class),"out of",length(unique(upreg)),"\n")
  cat("Downregulated genes:",length(downreg.class),"out of",length(unique(downreg)),"\n")
  cat("Binomial test p-value: ",prop.test(c(length(upreg.class),length(downreg.class)),c(length(unique(upreg)),length(unique(downreg))))$p.value,"\n \n")
}

# report on individual classes, excluding "other" and "unique":

with(palette[palette$Class != "other" & palette$Class != "unique",], for (i in 1:length(Class)) dist (dat2,Class[i]))
```

```
## Gene class: exocrine 
## Upregulated genes: 3 out of 1589 
## Downregulated genes: 12 out of 1505 
## Binomial test p-value:  0.02949373 
##  
## Gene class: classical 
## Upregulated genes: 8 out of 1589 
## Downregulated genes: 0 out of 1505
```

```
## Warning in prop.test(c(length(upreg.class), length(downreg.class)),
## c(length(unique(upreg)), : Chi-squared approximation may be incorrect
```

```
## Binomial test p-value:  0.01630234 
##  
## Gene class: RAS 
## Upregulated genes: 59 out of 1589 
## Downregulated genes: 29 out of 1505 
## Binomial test p-value:  0.003989242 
##
```
